# The impact of circadian rhythm disruption on oxaliplatin tolerability and pharmacokinetics in *Cry1*^*−/−*^*Cry2*^*−/−*^ mice under constant darkness

**DOI:** 10.1007/s00204-025-03968-7

**Published:** 2025-02-04

**Authors:** Yasemin Kubra Akyel, Narin Ozturk Seyhan, Şeref Gül, Melis Çelik, Ali Cihan Taşkın, Christopher P. Selby, Aziz Sancar, Ibrahim Halil Kavakli, Alper Okyar

**Affiliations:** 1 Department of Medical Pharmacology, School of Medicine, Istanbul Medipol University, Istanbul, Türkiye; 2 Department of Pharmacology, Faculty of Pharmacy, Istanbul University, TR-34116 Beyazit-Istanbul, Türkiye; 3 Biotechnology Division, Department of Biology, Faculty of Science, Istanbul University, Istanbul, Türkiye; 4 Department of Molecular Biology and Genetics, Koc University, Istanbul, Türkiye; 5 Present Address: Department of Laboratory Animal Science, Aziz Sancar Institute of Experimental Medicine, Istanbul University, Istanbul, Türkiye; 6 Present Address: Department of Biochemistry and Biophysics, School of Medicine, University of North Carolina, Chapel Hill, NC USA; 7 Department of Chemical and Biological Engineering, Koc University, Istanbul, Türkiye; 8 Present Address: Institute of Life Sciences and Biotechnology, Bezmialem Vakif University, Istanbul, Türkiye; 9 Translational Medicine Research Center, Experimental Animals Laboratory, Koc University, Istanbul, Türkiye

**Keywords:** Oxaliplatin, Chronopharmacokinetics, Chronotoxicity, Cryptochrome double-knockout mice, Circadian clock

## Abstract

Circadian rhythms, the 24-h oscillations of biological activities guided by the molecular clock, play a pivotal role in regulating various physiological processes in organisms. The intricate relationship between the loss of circadian rhythm and its influence on the tolerability and pharmacokinetic properties of anticancer drugs is poorly understood. In our study, we investigated the effects of oxaliplatin, a commonly used anticancer drug, on *Cry1*^*−/−*^ and *Cry2*^*−/−*^ mice (*Cry* DKO mice) under darkness conditions, where they exhibit free-running phenotype. We administered oxaliplatin at a dosage of 12 mg/kg/day at two distinct circadian times, CT8 and CT16, under constant darkness conditions to *Cry* DKO mice and their wild type littermates. Our results revealed a striking disparity in oxaliplatin tolerance between *Cry* DKO mice and their wild-type counterparts. Oxaliplatin exhibited severe toxicity in *Cry* DKO mice at both CT8 and CT16, in contrast to the wild type mice. Pharmacokinetic analyses suggested that such toxicity was a result of high concentrations of oxaliplatin in the serum and liver of *Cry* DKO mice after repeated dose injections. To understand the molecular basis of such intolerance, we performed RNA-seq studies using mouse livers. Our findings from the RNA-seq analysis highlighted the substantial impact of circadian rhythm disruption on gene expression, particularly affecting genes involved in detoxification and xenobiotic metabolism, such as the *Gstm* gene family. This dysregulation in detoxification pathways in *Cry* DKO mice likely contributes to the increased toxicity of oxaliplatin. In conclusion, our study highlights the crucial role of an intact molecular clock in dictating the tolerability of oxaliplatin. These findings emphasize the necessity of considering circadian rhythms in the administration of anticancer drugs, providing valuable insights into optimizing treatment strategies for cancer patients.

**Supplementary Information:**

The online version contains supplementary material available at 10.1007/s00204-025-03968-7.

## Introduction

Most species’ behavior and physiological changes are governed by the daily dark/night cycle are called circadian rhythm. These cyclic changes, have a period of approximately 24 h, are controlled by suprachiasmatic nuclei (SCN), located within the hypothalamus region of the mammals (Kavakli and Sancar 2002; Roenneberg and Merrow 2016). Circadian rhythm can be synchronized by environmental factors such as light (Roenneberg et al. 2003). Several diseases are associated with disruption of circadian rhythm such as metabolic disorders, sleep, and mood disorders (Allada and Bass 2021; Baris et al. 2023; Kavakli et al. 2022). Circadian rhythm is generated by a circadian clock through a transcriptional and translational feedback loop (TTFL) as a result of the interaction between core clock proteins (Kavakli et al. 2017; Partch et al. 2014; Takahashi 2017). In mammals, CLOCK and BMAL1 interact and then bind to the E-box region within the promoter region to initiate the transcription of clock-controlled genes, including periods (*Per*s) and cryptochromes (*Cry*s). Over the time, these two proteins accumulate in the cytosol and translocate to the nucleus, where they repress CLOCK/BMAL1-driven transcription (Kavakli et al. 2022; Takahashi 2017). Additional auxiliary proteins and post-translational modifications of core clock proteins modulate the TTFL and, in turn, control circadian clock at the molecular level (Rosensweig and Green 2020; Takahashi 2017).

Several studies indicate that the tolerability of anticancer drugs is under the control of circadian regulation (Dallmann et al. 2016; Levi et al. 2010, 2024; Okyar et al. 2024; Ozturk et al. 2017). Although it is not clear exactly how this is achieved at the molecular level, it has been suggested that rhythmic expression of genes that code for enzymes involved in metabolism and detoxification in different tissues may contribute to such effects (Dibner et al. 2010). Among the anticancer drugs studied, oxaliplatin has been widely investigated in terms of efficacy and pharmacokinetics when administered based on the time of the day (Amiama-Roig et al. 2022; Dallmann et al. 2016; Levi et al. 2010).

The efficacy of time-of-day specific delivery of anticancer drugs has given inconsistent data from different groups and as a consequence currently, there are no FDA approved chronochemotheraphy regimens (Sancar and Van Gelder 2021). We wished to investigate the pharmacodynamics and pharmacokinetics of the most widely used oxaliplatin drug in wildtype and genetically circadian disrupted mice to gain deeper insight into the effect of the circadian clock on the toxicity as a function of circadian time to provide a mechanistic foundation for optimal timing for administering oxaliplatin. We performed experiments using *Cry1*^*−/−*^*Cry2*^*−/−*^ mice (*Cry* DKO) and their wild type littermates, exposing them to oxaliplatin at two distinct circadian timepoints: CT8 and CT16, under constant darkness. Our primary objectives were twofold: to investigate the tolerance of these mice to oxaliplatin and to conduct transcriptomic analyses of their liver tissues to explore the underlying effects of chronomodulation in response to oxaliplatin treatment. *Cry* DKO animals exhibited lower tolerability compared to wild type animals at both CTs when administered oxaliplatin at a dose of 12 mg/kg/day, either as a single or repeated dose based on body weight changes. We also determined the pharmacokinetic (PK) profile of oxaliplatin using wild type and *Cry* DKO animals in serum and liver samples at both CTs. The results revealed that *Cry* DKO animals had altered PK profiles compared to wild type animals, with a notably increased clearance rate in *Cry* DKO animals at CT16. To gain insights into the molecular mechanisms underlying the circadian influence on oxaliplatin intolerance in *Cry* DKO mice with altered PK profiles, we performed transcriptomic analyses on liver tissue samples. The analyses indicated significant alterations in genes involved in detoxification such as *Gstm2*, *Gstm3*, *Gstm7*, and *Gstt1*.

Collectively, our findings highlight the significance of an intact molecular clock in minimizing the toxicity of oxaliplatin. This protection appears to be mediated through the regulation of genes responsible for detoxification processes. These insights into the circadian influence on tolerance may have implications for optimizing treatment schedules and minimizing adverse effects in cancer patients undergoing oxaliplatin therapy.

## Materials and methods

### Animals and synchronization

*Cryptochrome (Cry) 1/2* heterozygous mice in C57BL/6 J background were generously provided by Aziz Sancar (University of North Carolina, Chapel Hill, North Carolina, USA) and used to obtain *Cryptochrome* double-knockout (*Cry1*^*−/−*^*Cry2*^*−/−*^, DKO) male mice. The animals were kept in Koc University Research Center for Translational Medicine (KUTTAM) and genotyping was done to determine the knockouts. Wild type male littermates were used as control.

Animals (12–16 weeks old) were housed under 12 h of light- 12 h of dark cycle (LD 12:12) and synchronized for 3 weeks before the beginning of experiments. The mice were kept in constant darkness (DD) throughout the experiments, starting 3 days before the drug administration. All mice received a standard diet and food and water were provided ad libitum. Dim red light (7 lx) was used for experimental procedures during the dark phase.

### Experimental design

Oxaliplatin (Alfa Aesar, Germany) was diluted in 5% dextrose solution to a final dose of 12 mg/kg before each injection. The experiments were conducted in two arms: single-dose study and repeated-dose study. For single-dose study, oxaliplatin was intraperitoneally injected to the wild-type and *Cry* DKO male mice as a single dose at CT8 and CT16. The blood samples were collected under isoflurane anesthesia by cardiac puncture at 0.5, 2, 8 and 24 h after drug administration (*n* = 3–4 for each time point). After cervical dislocation, the livers were quickly removed and snap-frozen in liquid nitrogen. The blood samples were collected in EDTA tubes and the plasma was isolated by centrifugation at 4000 rpm for 10 min. Plasma and liver levels of oxaliplatin were determined by ICP-OES (Inductively coupled plasma-optical emission spectrometry) method as described below.

For repeated-dose study, oxaliplatin (12 mg/kg, i.p.) was given to wild-type and *Cry* DKO male mice at two circadian times (CT8, CT16) for three consecutive days. Body weight was measured every day as an index of general toxicity. Oxaliplatin-induced body weight change was expressed relative to body weight on the initial treatment day. The mice were sacrificed by cervical dislocation 24 h after 3rd injections (*n* = 4 for each CT). Blood and liver samples were collected. Hematological evaluation was performed in the blood samples. The liver samples were snap-frozen in liquid nitrogen and stored at − 80 °C until RNA extraction.

### RNA isolation and library preparation

Total RNA from liver samples (WT and CRYDKO mice treated with oxaliplatin at CT8 and CT16) were isolated by using RNeasy Mini Kit (QIAGEN, Germany). The quality of amount of RNA was assessed by using Qubit (Thermo Fisher Scientific, USA). 1 µg high quality RNA samples (OD_260/280_ 1.8–2.0) were used to prepare the library for RNA Sequencing analysis. Briefly, adenylated mRNAs were isolated by using Poly(A) mRNA Magnetic Isolation Module (New England Biolabs, USA). Then mRNAs were fragmented, converted to cDNA and second strand synthesis was performed. The 150 bp paired-end sequencing of libraries was carried out via Illumina platform (Macrogen, Netherlands).

### Transcriptome analysis

We followed a similar protocol, with some differences, that was published before (Cavga et al. 2019; Emisoglu-Kulahli et al. 2021). Briefly, the quality of sequencing reads was evaluated by using FastQC (Babraham Bioinformatics, UK) program. Trimmomatic (v0.35) was used to clean low-quality bases and adaptor contaminations (Bolger et al. 2014). Star aligner program (v2.5.3) was used to align clean reads to reference mouse genome (GRCm39). To determine differentially expressed genes (DEGs) among genetically different mice and time points, DESeq2 function (adjusted *p* < 0.05) in R was used (Love et al. 2015). Briefly, sorted reads by the STAR aligner were counted by using HTSeq (htseq-count v. 1.99.2) (Anders et al. 2015). Then, DEGs were calculated by using DESeq2 (Love et al. 2014). Low read counts were filtered by removing total read counts less than 10 across the independent biological samples. Variance stabilizing transformation was used by using vst function in DESeq2 to construct distance-distance matrix and to perform principal component analysis (PCA). Finally, statistically significant fold changes were calculated for genes having *p*-adjusted values < 0.05. To determine the significantly altered KEGG pathways in which DEGs were involved, ClueGo extension of Cytoscape (3.9.1) was used.

### Measurement of oxaliplatin concentrations in mouse plasma and liver

#### Chemicals

High-purity nitric acid was used in the ICP-EOS study (Merck, Istanbul, Türkiye). The purity of nitric acid was improved using the Berghof-Acid apparatus additionally. A standard solution of 1000 mg/L concentration was used for the platinum (VHG Labs, Manchester NH, USA). All standard solutions were prepared with ultrapure water obtained using the Elga Ultra-Pure System (Elga, UK). The standard solutions for measurement were prepared by appropriate dilution of these stock solutions on the day of use and stored in high-density polyethylene (HDPE) containers. Pure argon (99.999%) and pure nitrogen (99.99%) were supplied by Habaş, Türkiye. In the study, no glassware was used and all plastic materials were immersed in nitric acid (10%) for at least 24 h to significantly reduce contamination and were thoroughly rinsed with ultrapure water 3 times before use.

#### Microwave wet decomposition

Determining platinum levels with ICP-OES requires digesting tissue samples into a solution form prior to measurements. First, tissue samples were tared and dry-weighed with heat-resistant plastic ware tubes. Plasma samples were used directly as 100 µl. Prior to being microwaved, tissue samples were homogenized using ultrapure water with a tissue disintegration system (Magna Lyser, Roche, USA) and lyophilized at − 55 °C. 1.9 ml of nitric acid and 3 ml of ultrapure water were added to 100 µl plasma samples and placed in a microwave wet decomposition instrument (Berghoff, Italy) Teflon container. On the other hand, 3 ml of nitric acid and 2 ml of ultrapure water were added to tissue samples, weighed to 100 mg following lyophilization, and placed in Teflon containers. Microwave device parameters: Acid extraction was performed on the samples by applying 130 °C 35 bar for 8 min; 155 °C 35 bar for 5 min with 5 °C increments; 185 °C 35 bar for 12 min with 5 °C increments, 100 °C 35 bar for 5 min with 5 °C increments, and 50 °C 35 bar for 5 min with a sudden decrease.

#### ICP-OES parameters

For plasma and tissue samples, quantitative analyses of the platinum element were performed on a Perkin Elmer brand Optima 7000DV model ICP-OES (inductively coupled plasma–optical emission spectrometry) instrument using a concentric nebulizer, standard baffled cyclonic spray chamber, alumina injector, and quartz torch. The relative standard deviation was accepted as 10% and analyses with 9 replicates were performed. Instrument precision and accuracy analyses were performed using 0.6, 0.8, 1.0, 2.0, 4.0, and 5.0 μg/L platinum and 1.0 μg/L internal standard (manganese) standards. Calibration was done just before the true samples were analyzed and the stability was determined. The analysis of the samples was started by analyzing the platinum calibration points (5 ppb–100 ppb) with internal standards added to estimate their LODs (Limit of Detection), precision, accuracy, repeatability, linearity, and dynamic range. Instrument parameters: RF power (kW) was 1.3; nebulizer gas flow rate (L min^−1^) was 0.6; auxiliary gas flow rate (L min^−1^) was 0.2; plasma gas flow rate (L min^−1^) was 16.0; sample aspiration rate (mL min^−1^) was 1.0; platinum element detection wavelengths (nm) were 265,945 and 214,423; argon flow was 8 bar; nitrogen flow was 5 bar. The instrumental detection limit is given in Table S1.

### Pharmacokinetic analysis

The pharmacokinetic parameters of oxaliplatin were calculated using the non-compartmental pharmacokinetic analysis method. Peak plasma concentration (C_max_) and time to reach peak concentration (t_max_) values were directly obtained from the concentration–time curves. The area under the plasma concentration–time curve from 0 to 24 h (AUC_0–24 h_) was calculated using the linear trapezoidal rule. The total area under the plasma concentration–time curve from 0 h to infinity (AUC_0–∞_) was calculated using the standard formula (Concentration at 24 h (C_24h_)/Elimination rate constant (k_el)_)_._ The elimination rate constant (k_el_) was calculated from the terminal points of the concentration–time curve. T_1/2_ was calculated using the formula ln2/k_el_. The apparent total clearance (Cl/F) was calculated using the formula Dose/AUC_total_. The apparent distribution volume (Vd/F) was calculated by the formula (Cl/F)/k_el_.

### Statistical analysis

The data were presented as means ± standard error of means (SEM). Statistical analyses were performed using GraphPad Prism 5.00 (GraphPad Software, USA). The statistical significance of differences between groups was evaluated using Student’s *t*-test. Statistical significance required a *p* < 0.05.

## Results

### Toxicity of oxaliplatin in wild type and ***Cry1***^***−/−***^***Cry2***^***−/−***^ knockout mice depending on the circadian dosing time

**Fig. 1:**
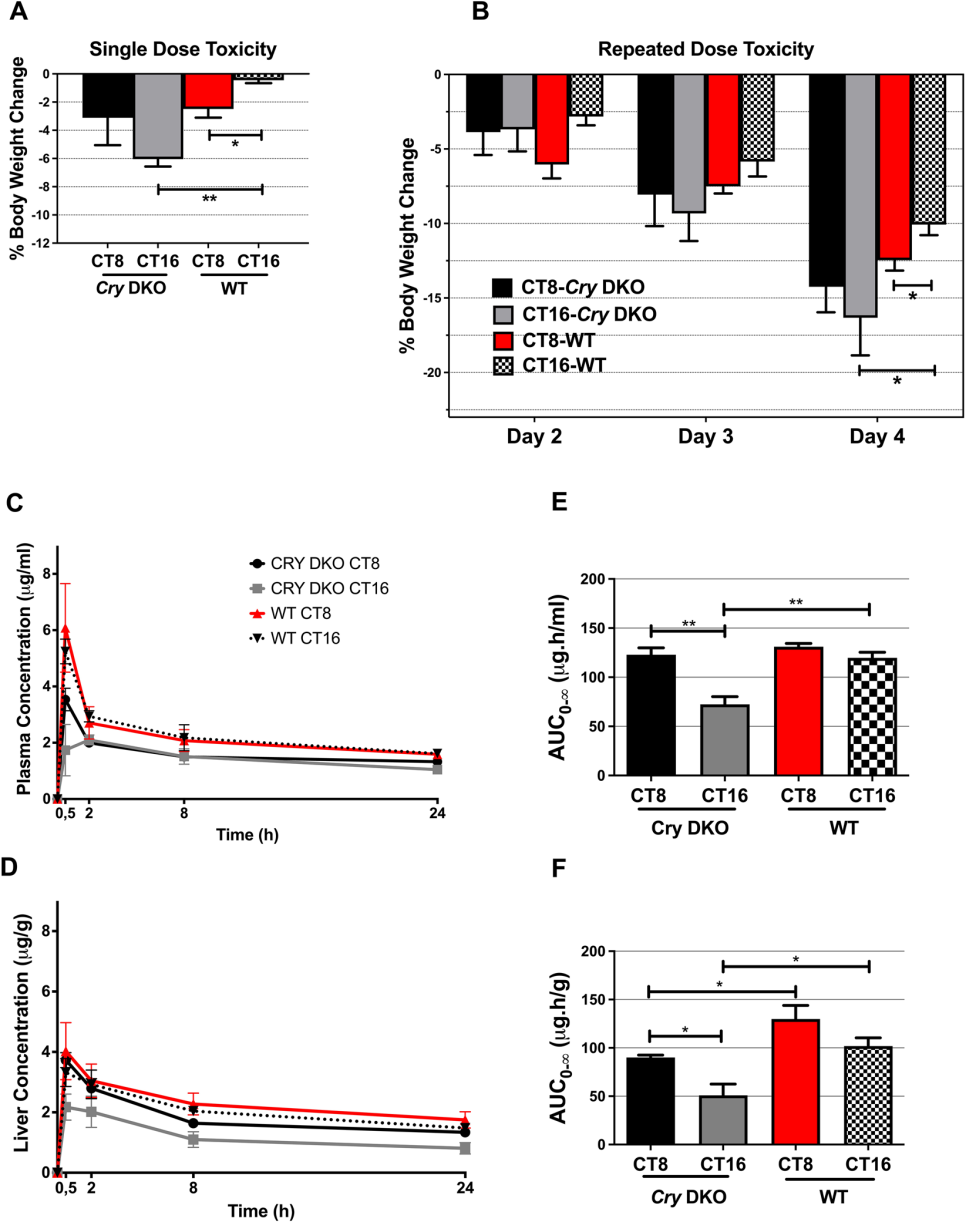
Toxicology and pharmacokinetic profile of oxaliplatin in animals. % Body weight change following single dose (**A**) or repeated dose (**B**) injection of oxaliplatin (12 mg/kg/day) to the *Cry* DKO and wild type mice at CT8 and CT16. The results are presented as mean ± SEM (*n* = 3–4) (*t*-test; *p < 0.05; **p < 0.01). Plasma and liver pharmacokinetics of oxaliplatin following 12 mg/kg single dose injection to the *Cry* DKO and wild type mice at CT8 and CT16. (**C**) Plasma concentration–time curve. **(D)** Liver concentration–time curve. (**E)** Plasma AUC_0–∞_ values. (**F)** Liver AUC_0–∞_ values. The results are presented as mean ± SEM (*n* = 3–4) (Student’s *t*-test; *p < 0.05; **p < 0.01)

To understand the effect of circadian rhythm on pharmacokinetic parameters, we generated *Cry1*^*−/−*^*Cry2*^*−/−*^ knockout mice (*Cry* DKO). We then placed *Cry* DKO animals in constant darkness, where they exhibit arrhythmic phenotype (Thresher et al. 1998; Vitaterna et al. 1999). To evaluate the toxicity of oxaliplatin on animals under constant darkness conditions, we administered a single dose of oxaliplatin at 12 mg/kg to both wild-type (WT) and Cry DKO animals at CT8 and CT16. First, general toxicity was assessed based on mortality, body weight changes, clinical signs, and hematological parameters. No mortality and clinical signs were observed in study groups of wild type and *Cry* DKO animals. Compared to WT mice, body weight loss (%) was significantly higher in the *Cry* DKO mice at CT16 (**p < 0.01). Dosing time-dependent body weight change was shown in WT mice, indicating significantly higher body weight loss at CT8 (*p < 0.05) (Fig. 1A).

Since *Cry* KO mice are more sensitive to oxaliplatin toxicity than WT mice, we reduced the dose by approximately 30%, resulting in a final 12 mg/kg dose. This was determined to be the maximum tolerated dose (MTD) for Cry DKO mice, ensuring the study could proceed without unacceptable toxicity. In the repeated dose study, oxaliplatin (12 mg/kg/day) was injected in three consecutive doses to WT and *Cry* DKO mice at CT8 and CT16. We evaluated the toxicity of the oxaliplatin by determining the body weight changes in all animals for 4 days. Overall, oxaliplatin was found to have an increasing toxic effect in all animals for each day of administration at all CTs (Fig. 1B). The mean body weight loss analyses indicated that oxaliplatin has similar toxic effect in *Cry* DKO animals, independent of CT. Although the toxicity of oxaliplatin increased in both CTs in WT animals, it exhibited a more pronounced effect at CT8 compared to CT16 on the 4th day (Fig. 1B). Similar to the single dose injections, after three repeated injections at CT16, oxaliplatin showed more toxicity in the *Cry* DKO animals, compared to the WT mice (*p < 0.05).

Collectively, our results suggested that repeated doses of oxaliplatin are toxic to animals at all CTs. Despite the more severe toxicity being observed in *Cry* DKO mice at all CTs, the degree of toxicity differed in wild type animals when administered at CT8 and CT16. These differences among different CTs in wild type mice might be attributed to the presence of an intact molecular circadian clock.

### Plasma and liver pharmacokinetics of oxaliplatin in wild type and *Cry1*^*−/−*^*Cry2*^*−/−*^ knockout mice depending on the circadian dosing time

**Fig. 2:**
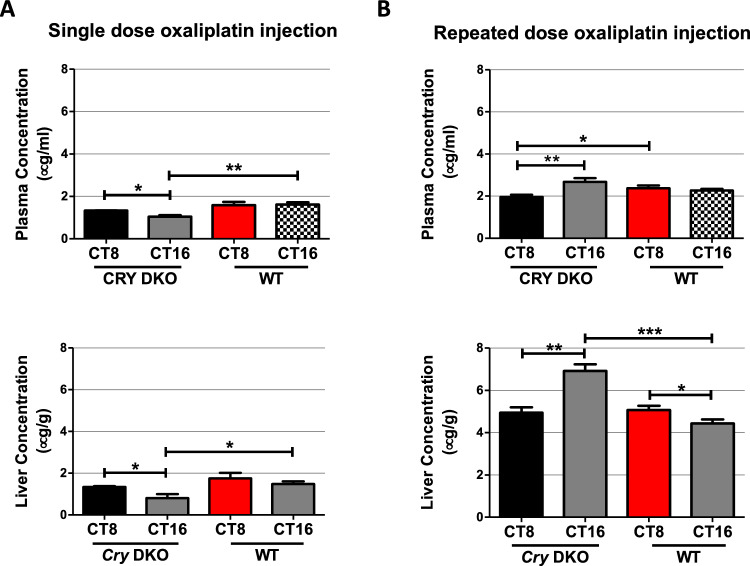
Plasma and liver concentrations of oxaliplatin at 24 h following 12 mg/kg single dose injection (**A**) and three repeated doses (**B**). Results are presented as mean ± SEM (*n* = 5) (Student’s *t*-test; *p < 0.05; **p < 0.01, ***p < 0.001) (*n* = 3–4)

We then investigated pharmacokinetic (PK) parameters of oxaliplatin in plasma and liver to assess the presence or absence of the molecular circadian clock influences tolerance to oxaliplatin. The results showed that pharmacokinetic parameters of oxaliplatin differed in plasma and liver depending on the circadian time of drug administration in both WT and *Cry* DKO animals (Table 1). The maximum plasma levels were reached within 30 min in all groups except for *Cry* DKO given oxaliplatin at CT16, which had a t_max_ of 2 h. Subsequently, oxaliplatin plasma levels declined with apparent half-lives (t_1/2_) ranging from 25 to 44 h. Oxaliplatin was still detectable 24 h after administration with a relatively long t_1/2_ in *Cry* DKO mice when dosed at CT8 compared to CT16 (*p < 0.05). The mean C_max_ values of oxaliplatin in WT mice were 1.7-fold and 2.1-fold higher than those in *Cry* DKO mice when the drug dosed at CT8 and CT16, respectively (*p < 0.05). The mean plasma concentration–time curve of oxaliplatin in WT and *Cry* DKO mice is given in Fig. 1C**.** When the AUC_0-∞_ values in *Cry* DKO mice were compared based on the administration time, the mean AUC_0-∞_ value of oxaliplatin at CT8 was found to be 1.7-fold higher than at CT16 (**p < 0.01). Plasma AUC_0–∞_ values of oxaliplatin were comparable at CT8 and CT16 in WT mice (Fig. 1C). The evaluation of the oxaliplatin AUC_0–∞_ values based on the circadian genotype revealed that plasma AUC_0–∞_ values in *Cry* DKO and WT mice were comparable at CT8, whereas a significant difference in AUC_0–∞_ values between *Cry* DKO and WT mice occurred at CT16 (**p < 0.01). The mean concentration–time curve of oxaliplatin in the liver for wild type and *Cry* DKO is given in Fig. 1D. The mean C_max_ values of oxaliplatin in plasma and liver were significantly higher in *Cry* DKO mice when dosed at CT8 compared to CT16 (*p < 0.05). Liver AUC_0-∞_ values after single-dose oxaliplatin administration were comparable to plasma. Liver AUC_0–∞_ values of oxaliplatin in WT mice were 1.4-fold and twofold higher than those in *Cry* DKO mice at CT8 and CT16, respectively (*p < 0.05), indicating a higher exposure to the oxaliplatin in WT mice (Fig. 1D). When time-dependent pharmacokinetic variations were assessed in the *Cry* DKO mice liver, the mean liver AUC_0–∞_ value of oxaliplatin at CT8 was found to be 1.8-fold higher than at CT16 (*p < 0.05). The mean plasma clearance (CL) of oxaliplatin in *Cry* DKO mice at CT16 was about twofold higher than at CT8 (*p < 0.05). Furthermore, the mean CL value of oxaliplatin in plasma was higher in *Cry* DKO mice dosed at CT16 compared to WT mice (*p < 0.05). Plasma and liver oxaliplatin levels after 24 h and three-repeated oxaliplatin administration were shown in Fig. 2.

**Table 1:** Pharmacokinetic parameters of oxaliplatin in plasma and liver were assessed following a single dose injection of 12 mg/kg to both *Cry1*^*−/−*^*Cry2*^*−/−*^ knockout and wild-type mice at CT8 and CT16

Pharmacokinetic parameters	*Cry1*^*−/−*^*Cry2*^*−/−*^ knockout		Wild type	
	CT8	CT16	CT8	CT16
Plasma				
AUC_0–24 h_ (μg h/ml)	38.13 ± 1.97	34.63 ± 4.80^#^	51.79 ± 8.93	53.20 ± 6.84
AUC_0–∞_ (μg h/ml)	122.93 ± 6.96*	72.42 ± 7.77^#^	131.07 ± 3.36	119.69 ± 5.79
C_max_ (μg/ml)	3.54 ± 0.40*	2.10 ± 0.30^#^	6.08 ± 1.57^£^	5.25 ± 0.44
t_max_ (h)	0.5	2	0.5	0.5
t_1/2_ (h)	44.36 ± 4.89*	24.78 ± 4.34	36.11 ± 7.84	28.70 ± 2.80
CL (ml/h)	1.96 ± 0.11*	3.39 ± 0.37^#^	1.83 ± 0.05	2.02 ± 0.10
V_d_ (ml)	124.24 ± 6.74	118.70 ± 15.86	95.26 ± 20.73	83.81 ± 10.64
Liver				
AUC_0–24 h_ (μg h/g)	42.93 ± 3.18	28.26 ± 6.68^#^	54.43 ± 8.91	48.57 ± 4.44
AUC_0–∞_ (μg h/g)	90.13 ± 2.54*	50.86 ± 11.65^#^	129.86 ± 19.77^£^	101.88 ± 8.55
C_max_ (μg/g)	3.73 ± 0.25*	2.18 ± 0.44	4.02 ± 0.95	3.32 ± 0.46
t_max_ (h)	0.5	0.5	0.5	0.5
t_1/2_ (h)	24.72 ± 3.40	19.24 ± 2.34	30.03 ± 2.31	25.09 ± 2.82

The results are presented as mean ± SEM (*n* = 4)

t_max_: The time when the maximum concentration is reached. C_max_: The maximum concentration. t_1/2_: Elimination half-life. AUC_0–24 h_: Area under the curve between 0 and 24 h. AUC_0-∞_: Area under the total curve between 0 to infinity. Cl: Clearance. Vd: Distribution volume.

^*^p < 0.05 *Cry* DKO CT8 vs *Cry* DKO CT16,

^#^*p* < 0.05 *Cry* DKO CT16 vs WT CT16,

^£^*p* < 0.05 *Cry* DKO CT8 vs WT CT8

### RNA-seq analysis

**Fig. 3:**
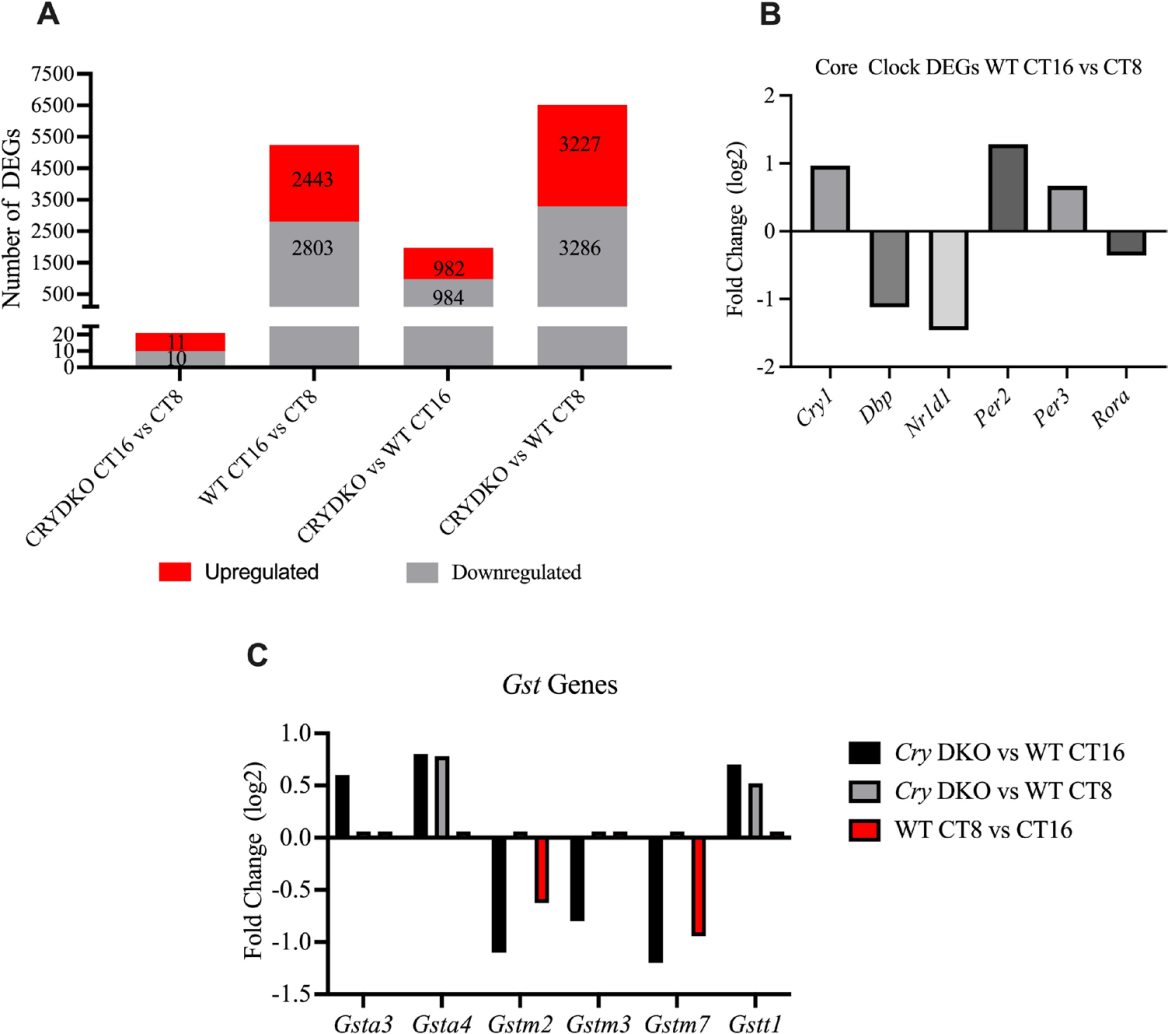
Analysis of RNA-Seq data from Cry double knockout and their wildtype littermate. **A** The number of DEGs obtained after the comparison of different genetic background mice (WT or *Cry* KO) at different time points (CT8 or CT16). **B** Fold change of core clock genes obtained from the DEG analysis between WT mice at CT16 vs CT8. **C** Fold change of *Gst* genes obtained from the DEG analysis between WT vs *Cry* DKO mice at CT16; WT vs *Cry* DKO mice at CT8; and WT mice at CT16 vs CT8

The toxicity and pharmacokinetic studies after repeated dose injections of oxaliplatin suggest that oxaliplatin stays in the liver for a long period of time and results in enhanced toxicity in *Cry* DKO mice. We hypothesized that disruption of the circadian clock by knocking out *Cry1/2* animals may result in disruption of transcriptional regulation of oxaliplatin metabolizing enzymes and, in turn, increase the half-life of oxaliplatin. To this end, we performed a genome-wide study to identify differentially regulated genes by RNA-seq. We isolated total RNA from the liver of wild type and *Cry* DKO mice at CT8 and CT16. After preparation of the libraries, we performed RNA-Seq analysis. The clean reads were mapped to the mouse genome (GRCm39) by using the STAR aligner program. The total reads of all samples were given in Figure S1A. DESeq2 package of R programming was used to assess the quality of our reads and to determine the set of differentially expressed genes (DEGs) (Love et al. 2015). Since DESeq2 needs read counts, we used HTSeq to count the reads (Anders et al. 2015), followed by quality control using log2 transformed counts obtained from the variance stabilizing transformation (VST) (Anders and Huber 2010; Tibshirani 1988). The primary goal of utilizing VST is to eliminate the variance's dependence on the mean, especially when log counts with low means have large variances. Here, by “quality control”, we mean how different samples are separated from each other, or in other words, how “similar” and “dissimilar” they are. Thus, we constructed a distance matrix among samples by using the transformed data (Fig. S1B). The distance matrix showed that wild type samples at CT8 and CT16 are far apart from each other, as expected, since they are collected at two different circadian phases. In contrast, *Cry* KO liver samples at CT8 and CT16 were not distantly apart, providing evidence that gene expressions in mutant mice were similar at different CTs, because the animals are arrhythmic under constant darkness. Overall, WT and *Cry* DKO mice samples are far away from each other. We also performed principal component analysis (PCA) of the transformed data to show similarities and dissimilarities of samples. PCA allows to investigation of the overall impact of experimental factors and batch effects on a 2D plane (Fig. S1C). As can be seen from Fig. S1B and C, WT samples are separated at two different circadian times, while no such effect was observed on *Cry* DKO mice samples. We continued with DEG analysis by using DESeq2 package at interested time points and conditions. In DEG analysis only significantly changed genes (adjusted *p* < 0.05) were reported. We initially analyzed the DEGs among WT mice at CT16 and CT8. Totally 5246 DEGs were determined where 2443 genes were upregulated, and 2803 genes were downregulated (Fig. 3A). We next analyzed the transcription levels of core clock genes whose transcription is under the control of the circadian clock in both WT and *Cry* DKO animals (Fig. 3A). While core clock genes showed significantly different expression levels at two CTs in wild type mice, no such regulation was observed in *Cry* DKO mice. Differential expression of clock-controlled genes at two different CTs demonstrates the distinct phases of WT animals. KEGG pathway analysis from these DEGs showed that 18 pathways were significantly affected (Table S2).

Next, we investigated DEGs among WT and *Cry* DKO mice samples at CT16, where the highest difference in toxicity was observed. A total of 1966 DEGs were detected where detected with 982 genes upregulated and 984 genes downregulated in *Cry* DKO samples (Fig. 3A**)**. Subsequent KEGG pathway analysis showed that seven pathways were significantly affected (Table 2). Especially, xenobiotics metabolism, drug metabolism (cytochrome P450 and other enzymes), and chemical carcinogenesis pathways that are likely to be involved in oxaliplatin metabolism were significantly changed. Inspection of these four pathways showed that glutathione S-transferases (GSTs) e.g. *Gsta3*, *Gsta4, Gstm2, Gstm3, Gstm7*, and *Gstt1* are commonly found. GSTs are critical enzymes for the detoxification process of cells by catalyzing the coupling of glutathione (GSH) with various xenobiotics such as 4-hydroxynonenal and cisplatin (Townsend and Tew 2003; Yang et al. 2016). We also calculated the DEGs between WT and *Cry* DKO mice at CT8, in which oxaliplatin exhibits no significant toxicity. The 6513 DEGs were determined among those samples in which 3227 were upregulated, 3286 were downregulated (Fig. 3A). KEGG pathway analysis from DEGs revealed that 16 pathways were significantly changed, however, none of them were related to drug metabolism (Table S3) and none of the GST family proteins were involved in these pathways. We further compared the fold change of *Gst* genes determined in the DEG analysis between WT vs *Cry* DKO mice at CT16, WT vs *Cry* DKO mice at CT8, and WT mice at CT16 vs CT8 (Fig. 3B). While *Gsta4* and *Gstt1* showed similar fold change in WT vs *Cry* DKO in both time points; *Gstm2, Gstm3*, and *Gstm7* were downregulated, and *Gsta3* was upregulated only at CT16 when CRY genes were knock-outed. Thus, we proposed that the higher toxicity of oxaliplatin stems from the differential regulation of *Gstm2, Gstm3, Gstm7* and *Gsta3*.

**Table 2:** KEGG pathway analysis of DEGs obtained from wild type and *Cry1*^*−/−*^*Cry2*^*−/−*^ knockout mice at CT16. Glutathione S-transferase (GST) genes are shown in bold

Term	Term *p*-value Corrected	Corrected group *p*-value	Associated genes (%)	Number of genes	Associated genes found
Pentose and glucuronate inter-conversions	3.45E−02	1.51E−04	31.4	11	Akr1b3, Dhdh, Ugdh, Ugp2, Ugt1a10, Ugt1a6a, Ugt1a6b, Ugt1a9, Ugt2b35, Ugt2b36, Xylb
Retinol metabolism	1.87E−04	1.61E−06	25.8	25	Adh1, Aldh1a7, Aox3, Bco1, Cyp1a2, Cyp26a1, Cyp2a5, Cyp2b10, Cyp2b13, Cyp2b9, Cyp2c23, Cyp2c37, Cyp2c40, Cyp3a25, Cyp4a10, Cyp4a14, Cyp4a32, Rdh16f2, Rdh9, Ugt1a10, Ugt1a6a, Ugt1a6b, Ugt1a9, Ugt2b35, Ugt2b36
Steroid hormone biosynthesis	1.03E−02	5.53E−08	22.8	21	Cyp1a2, Cyp2b10, Cyp2b13, Cyp2b9, Cyp2c23, Cyp2c37, Cyp2c40, Cyp2e1, Cyp3a25, Cyp7a1, Dhrs11, Hsd11b1, Hsd3b3, Srd5a2, Sts, Ugt1a10, Ugt1a6a, Ugt1a6b, Ugt1a9, Ugt2b35, Ugt2b36
Metabolism of xenobiotics by cytochrome P450	3.32E−03	5.53E−08	26.0	19	Adh1, Cyp1a2, Cyp2e1, Dhdh, **Gsta3, Gsta4, Gstm2, Gstm3, Gstm4, Gstm6, Gstm7, Gstt1**, Hsd11b1, Ugt1a10, Ugt1a6a, Ugt1a6b, Ugt1a9, Ugt2b35, Ugt2b36
Drug metabolism—cytochrome P450	5.28E−04	5.53E−08	28.2	20	Adh1, Aox3, Cyp1a2, Cyp2e1, Fmo2, Fmo3, **Gsta3, Gsta4, Gstm2, Gstm3, Gstm4, Gstm6, Gstm7, Gstt1,** Ugt1a10, Ugt1a6a, Ugt1a6b, Ugt1a9, Ugt2b35, Ugt2b36
Drug metabolism—other enzymes	6.29E−05	5.53E−08	27.2	25	Cda, Ces1d, Ces1f, Cyp2e1, **Gsta3, Gsta4, Gstm2, Gstm3, Gstm4, Gstm6, Gstm7, Gstt1,** Nat2, Nme4, Tpmt, Tymp, Uck1, Ugt1a10, Ugt1a6a, Ugt1a6b, Ugt1a9, Ugt2b35, Ugt2b36, Upb1, Upp2
Chemical carcinogenesis	3.05E−05	5.53E−08	26.7	27	Adh1, Cyp1a2, Cyp2b10, Cyp2b13, Cyp2b9, Cyp2c23, Cyp2c37, Cyp2c40, Cyp2e1, Cyp3a25, **Gsta3, Gsta4, Gstm2, Gstm3, Gstm4, Gstm6, Gstm7, Gstt1,** Hsd11b1, Kyat3, Nat2, Ugt1a10, Ugt1a6a, Ugt1a6b, Ugt1a9, Ugt2b35, Ugt2b36

Additionally, the number of DEGs among Cry DKO samples at two different CTs was 10 genes downregulated, and 11 genes upregulated, suggesting that KO animals lacked a discernible circadian rhythm.

## Discussion

The circadian rhythm, a fundamental biological process, plays a crucial role in regulating various physiological functions, including responses to cancer therapy (Levi et al. 2010). This rhythm is orchestrated by the molecular clock, which involves interactions among core clock proteins, ultimately driving circadian gene expression through transcriptional and translational feedback loops (TTFL) as results of interaction between core clock proteins (Kavakli et al. 2017). In this study, we aimed to elucidate how intact circadian rhythm influences the pharmacokinetics, and toxicity of the anticancer drug oxaliplatin. We used *Cry1*^*−/−*^*Cry2*^*−/−*^ (*Cry* DKO) mice, which lack a functional circadian rhythm (Thresher et al. 1998; Vitaterna et al. 1999), to investigate the impact of circadian disruption on oxaliplatin response under constant darkness conditions.

Our findings revealed that *Cry* DKO animals exhibited reduced tolerance to oxaliplatin, evidenced by greater body weight loss compared to wild type (WT) littermates (Fig. 1). This diminished tolerance was consistent across different dosing schedules and circadian times, highlighting the critical role of circadian rhythm in modulating drug response. Moreover, the absence of *Cry* genes led to a disruption in circadian toxicity patterns, further emphasizing the importance of an intact circadian clock for proper drug tolerability*.* The studies suggested that periodic transcriptional activation and repression of target genes by the CLOCK:BMAL1 transactivation complex causes circadian variations in the physiological processes of anticancer drug pharmacokinetics and pharmacodynamics (Lowrey and Takahashi 2004). In fact, a study shows that in vivo sensitivity to anticancer drug, cyclophosphamide (CY), depends on the functional status of the CLOCK/BMAL1 transactivation complex (Gorbacheva et al. 2005). *Bmal1* and *Clock* knockout mice showed sensitivity to CY while CRY knockout exhibited resistance against CY treatment under 12L:12D conditions (Gorbacheva et al. 2005) where mice express constantly high levels of CLOCK/BMAL1 transcriptional activity. Our observation of poorer oxaliplatin tolerability in *Cry* DKO compared to WT animals (Fig. 1A, B) can be explained by our experimental design, which involved conducting the study under constant darkness conditions, where *Cry* DKO mice lack a circadian rhythm.

WT mice treated at CT8 lost significantly more weight compared with the WT treated at CT16 (*p* < 0.05) similar to the previously reported results in male B6D2F1 mice (Boughattas et al. 1989). A body weight loss profile similar to that of a single dose was observed when oxaliplatin was administered to mice for 3 days (Fig. 1A, B). The dosing-time dependent difference in body weight loss increased with the repeated dose used. Interestingly, this circadian toxicity pattern was abolished in *Cry* DKO mice. Strikingly, single and repeated dose oxaliplatin administrations at CT16 (best tolerated time of the drug in WT mice) caused more apparent body weight loss in the absence of *Cry* genes as compared to their WTs (*p* < 0.01 and *p* < 0.05, respectively) (Fig. 1A, B). There is a critical notice in attempts to associate circadian clock function with physiological variation since all clockless animals are not equivalent (Sancar and Van Gelder 2021). As transcription factors, each circadian clock protein has specific targets that may be non-circadian, that results in a distinct pleiotropy when mutated (Koike et al. 2012). The state of the circadian clock in each KO may be distinct; for example, a *Bmal1* null animal may leave a gene in a chronically low expression state, whereas *Cry* DKO mutants may result in a high-expression state of the same transcript (Sancar and Van Gelder 2021). Time-dependent tolerability of the oxaliplatin derivative of cisplatin in male WT mice was studied, with the optimal time identified as CT16 in a DD (constant darkness) condition (Dakup et al. 2018). Mice treated in the evening exhibited a heightened rate of removal of cisplatin–DNA adducts and experienced less toxicity compared to those treated in the morning. This temporal discrepancy in toxicity was absent in mice lacking the *Per1* and *Per2* genes (*Per1*^*−/−*^* Per2*^*−/−*^), indicating that the time-of-day effect is intricately linked to the circadian rhythm. Further studies revealed that the chronopharmacology of cisplatin involves the circadian regulation of DNA repair mechanisms alongside immune responses. A recent study investigated formation and repair of damage from cisplatin administered to mice at 4 h intervals over a 24-h period (Yang et al. 2018). They found that the transcribed strand (TS) of DNA was preferentially repaired at a higher rate compared to the non-transcribed strand of DNA in a circadian-dependent manner. Considering the action mechanism of oxaliplatin is similar to cisplatin, it is possible that DNA damage caused by oxaliplatin was slowed down on the TS and caused severe toxicity on *Cry* DKO animals under constant conditions in all CTs. Therefore, it is difficult to generalize from the results of any single clock gene KO animal about the role of the circadian clock in the observed physiological function. However, it can be concluded that there is a mechanistic link between the circadian clock and oxaliplatin tolerability.

The plasma concentration–time profiles of experimental groups showed that oxaliplatin pharmacokinetics differed in terms of C_max_ and AUC_0-24 h_ between WTs and *Cry* DKO mice, regardless of the time of administration (Table 1). In single dose oxaliplatin study, plasma C_max_ and AUC_0–24 h_ values of oxaliplatin were higher in WT than in *Cry* DKOs. However, C_max_ and AUC_0–24 h_ of oxaliplatin in WT groups did not significantly differ according to dosing times i.e. CT8 and CT16. Interestingly, the lowest C_max_ and AUC_0–24 h_ values of oxaliplatin in plasma were detected in *Cry* DKO treated at CT16. Plasma C_max_ and AUC_0–24 h_ of oxaliplatin were higher in WT mice than in *Cry* DKOs, this difference was more pronounced in CT16. Our pharmacokinetic data partially supports the findings regarding the dosing time-dependent tolerability of the drug in WT mice. Higher C_max_ and AUC_0–∞_ values and longer elimination half-lives in plasma were observed in WT when treated at CT8 as compared to CT16. Peak concentrations, AUC_0–24 h_ and AUC_0–∞_ values of oxaliplatin in the liver in CT8-treated WT mice also showed similar concentration–time profiles as in plasma, supporting the drug tolerability data. However, a comparison of the plasma and liver concentration vs time profiles of oxaliplatin between two groups of WT animals with proper clock function revealed that the time of drug administration did not affect oxaliplatin exposure confirming the previously published findings in male B6D2F1 mice (Boughattas et al. 1994) and also supporting the lack of an apparent relationship between plasma PK and chronotoxicity.

In all experimental groups, no marked relationship was observed between body weight loss and drug exposure following single-dose drug administration. However, in *Cry* DKO mice administered repeated doses of oxaliplatin at CT16, the drug accumulated in the plasma and liver compartments for up to 24 h after discontinuation of oxaliplatin (Fig. 2). Correspondingly this group of mice experienced the greatest body weight loss (Fig. 1). Similarly, in the WT groups receiving repeated doses of oxaliplatin, the body weight loss, which was higher at CT8 than at CT16, correlated with the accumulated drug concentration in the body.

### Detoxification of oxaliplatin by GSTs

Sensitivity or resistance to chemotherapy and toxicity of anticancer agents are largely determined by detoxification of enzymes in the body. Differential function or expression of detoxifying enzymes may be involved in the differences in oxaliplatin concentrations between experimental groups in our current study. Oxaliplatin, like other platinum-derived drugs, is detoxified by the enzymes belonging to the glutathione *S*-transferases (GSTs) family in the body (Katayanagi et al. 2019; Kwon et al. 2007; Lecomte et al. 2006). The studies provide strong evidence of the direct involvement of the GSTP1 subclass in oxaliplatin detoxification and resistance to platinum compounds (Kweekel et al. 2009; Lecomte et al. 2006; Stoehlmacher et al. 2002). In addition to GSTP1, the activity of GSTM1 and GSTT1 may be partly implicated in the response to platinum-based cancer treatment and the detoxification of platinum compounds (Funke et al. 2010; Lecomte et al. 2006). Other studies also support the involvement of GSTM1 in the sensitivity to platinum-based agents (Funke et al. 2010; Kweekel et al. 2009; Stoehlmacher et al. 2002). The changes in gene expression of these enzymes which are associated with cellular platinum drug clearance, may affect oxaliplatin sensitivity by changing intracellular concentrations of the drug. Especially GSTP, and partly GSTM and GSTT isoenzymes participate in the detoxification of platinum compounds.

In our study, RNA-sequencing analysis performed 24 h after the last dose of oxaliplatin following three days of treatment indicated that dosing-time and *Cry*-dependent variations in oxaliplatin sensitivity and pharmacokinetics may be partly attributed to the variations in gene expressions related to oxaliplatin detoxification/metabolism. The repeated dose study displayed that detoxification of oxaliplatin was disrupted in *Cry* DKO mice, leading to increased drug exposure (Fig. 2). The upregulation in expression of *Gstm2, Gstm3*, and *Gstm7 genes* in WT mice, compared to mutant mice, treated at CT16, leading to increased enzyme activity, may have been related to enhanced detoxification of oxaliplatin and shortened exposition to the drug, and therefore, have been hypothesized to result in decreased sensitivity to oxaliplatin treatment in this group of mice.

## Conclusion

In conclusion, our study elucidated the critical role of the circadian rhythm in modulating the pharmacokinetics and toxicity of the anticancer drug oxaliplatin. We showed time-dependent toxicity of oxaliplatin was abolished in *Cry* DKO mice in our study. The liver mRNA expression of enzymes in which play a role of oxaliplatin detoxification and oxaliplatin-induced damage was also display no significant differences between CT8 and CT16. The loss of circadian rhythm in the body may cause deterioration in the drug metabolism/detoxification. Furthermore, our pharmacokinetic analyses revealed differences in oxaliplatin exposure between *Cry* DKO mice and WT animals, with *Cry* DKO mice exhibiting lower plasma and liver concentrations of the drug. RNA-sequencing analysis indicated that variations in oxaliplatin sensitivity and pharmacokinetics, influenced by dosing-time and *Cry*-dependent mechanisms, may be attributed to alterations in gene expressions related to oxaliplatin detoxification and metabolism. Specifically, upregulation of *Gstm2, Gstm3*, and *Gstm7* genes in WT mice, compared to mutant mice, treated at certain circadian times may lead to enhanced detoxification of oxaliplatin and reduced drug sensitivity.

These findings highlight the complicated relationship between the circadian clock and the pharmacological response to anticancer drugs. While further research is warranted to fully elucidate the underlying mechanisms, our study underscores the importance of considering circadian rhythms in the development and optimization of cancer therapies. Ultimately, a better understanding of circadian regulation in drug response may pave the way for personalized treatment strategies and improved outcomes for cancer patients. It is also important to recognize that the doses used in preclinical models, such as mice, are not directly translatable to human therapeutic doses due to differences in body surface area, metabolism, and physiology. In oncology research, anticancer drugs are typically administered at or near their maximum tolerated dose (MTD) to balance therapeutic efficacy with acceptable toxicity. Although the dose applied in this study may not align precisely with human therapeutic regimens, it adheres to standard preclinical practices and serves as a foundation for assessing drug efficacy and tolerability in this experimental model.

## Supplementary Information

Below is the link to the electronic supplementary material.

Supplementary file1 (DOCX 377 KB)

## Data Availability

All cleaned, high-quality RNA sequencing data were uploaded to NCBI Gene Expression Omnibus (GEO) database under the GEO Accession number GSE24349.
