# Supplementary material for: The impact of circadian rhythm disruption on oxaliplatin tolerability and pharmacokinetics in Cry1−/−Cry2−/− mice under constant darkness
Source: Arch Toxicol. 2025 Feb 4;99(4):1417–29. doi: 10.1007/s00204-025-03968-7 (PMC11968489; doi:10.1007/s00204-025-03968-7)
Supplement: Supplementary file 1 — Supplementary file1 (DOCX 377 KB) [file 204_2025_3968_MOESM1_ESM.docx]

**Figure S1: Analysis of RNA-Seq reads. A**) Read numbers of each replicate from WT and KO mice. **B**) Distance-to-distance matrix of all reads obtained from each sample. **C**) The principal component analysis of all reads.
